# Microwave ablation combined with attenuated *Salmonella typhimurium* for treating hepatocellular carcinoma in a rat model

**DOI:** 10.18632/oncotarget.17468

**Published:** 2017-04-27

**Authors:** Qing Zhao, Xudong Qu, Kai Liu, Huibin Shi, Guowei Yang, Bo Zhou, Liang Zhu, Wei Zhang, Zhiping Yan, Rong Liu, Sheng Qian, Jianhua Wang

**Affiliations:** ^1^ Department of Interventional Radiology, Zhongshan Hospital, Fudan University, Shanghai, 200032, China; ^2^ Department of Radiology, Zhongshan Hospital, Fudan University, Shanghai, 200032, China; ^3^ Shanghai Institute of Medical Imaging, Shanghai, 200032, China

**Keywords:** hepatocellular carcinoma, VNP 20009, microwave ablation

## Abstract

We aim to investigate the safety and efficacy of microwave ablation (MWA) combined with attenuated *Salmonella typhimurium* strain VNP20009 in treating hepatocellular carcinoma. Portions of tumor tissues were orthotopically implanted in the livers of 40 male rats weighed 150~200 g to establish tumor models. Three weeks later, the rats were randomly divided into four groups: (A) MWA plus VNP20009 group; (B) MWA group; (C) VNP20009 group; and (D) control group. Incomplete MWA was performed (20~30 W, 1~2 min) after the hepatic carcinoma was properly exposed. VNP20009 (about 1×10^7^ cfu) was directly injected into the tumor immediately. MRI scans were performed to assess the tumor responses 7 and 14 days later, respectively. Micro CT was used to observe the lung metastases. After the animals were sacrificed or died, the tumors were cut off for the purpose of pathological and immunohistochemical analyses. The results showed that the mean tumor volumes of MWA plus VNP20009 group on the 7th and 14th day post treatment were obviously smaller than those of other groups (P < 0.05). Lung metastases rates were 20%, 60%, 30% and 100% in MWA plus VNP20009 group, MWA group, VNP20009 group and control group, respectively. The median survival of the rats in MWA plus VNP20009 group was distinctly longer than those in other groups (P < 0.05). In summary, MWA combined with VNP20009 produced better effects than MWA or VNP20009 alone in treating hepatic carcinoma. This strategy might have potential ability to decrease lung metastases and prolong the overall survival.

## INTRODUCTION

Hepatocellular carcinoma (HCC) is one of the most common human malignancies with high mortality rate in developing countries [1]. Thermal ablations (microwave ablation and radiofrequency ablation) with the advantages of minimal invasiveness, effectiveness, lesser technical demand, and repeatability, have been widely used in the treatment of HCC [2, 3]. However, complete necrosis is difficult to achieve for thermal ablation therapy in tumors larger than 3 cm in diameter or difficult to access. The remaining viable tumor cells have a great impact on the prognosis and long-term survival of patients. Thus, combinatory therapy is supposed to be an hopeful strategy to improve the efficacy of thermal ablation. For example, thermal ablation combined with systemic chemotherapy or transarterial chemoembolization have been proved to be feasible, safe and effective [4–7].

It was in 1893 that anaerobia were for the first time used for cancer treatment [8]. Certain species of anaerobia, such as *Salmonella typhimurium*, *bifidobacterium*, and *Clostridium*, had been proved to have antitumor activity [9–14]. VNP20009 is a genetically engineered *Salmonella typhimurium* strain. As a result of deleting the *msbB* and *purI* genes, it is not able to produce endotoxin. VNP20009 had been deployed as a potent anticancer agent in multiple animal tumor models, and its safety profiles had been demonstrated in previous phase 1 clinical trials [12, 15, 16]. However, to our knowledge, its application in the treatment of HCC remains to be explored. In this study, we aim to evaluate the applicability and efficacy of microwave ablation (MWA) combined with VNP20009 in treating HCC in a rat model.

## RESULTS

### *In vitro* experiments

**Figure 1:**
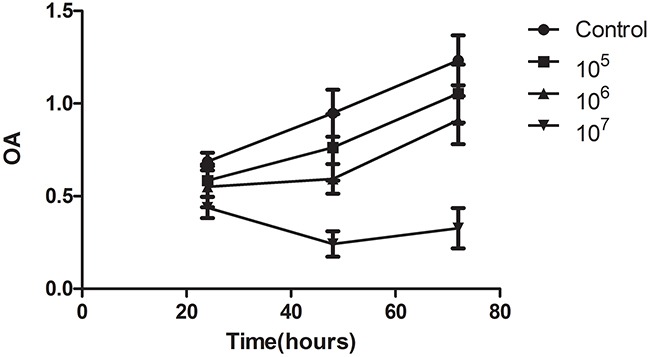
VNP 20009 inhibits the proliferation of McA-RH7777 cells The OD values of VNP 20009 groups (10^5^, 10^6^, 10^7^ per well) were obviously lower than those of control groups and decreased as the bacteria concentration increased.

**Figure 2:**
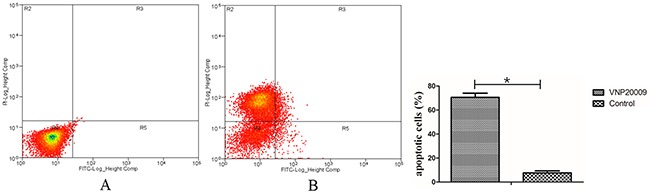
VNP 20009 induces apoptosis of McA-RH7777 cells **(A)** Control group; **(B)** VNP 20009 group. The percentage of apoptic cells in Control group was much higher than that in VNP 20009 group (* P < 0.05).

**Figure 3:**
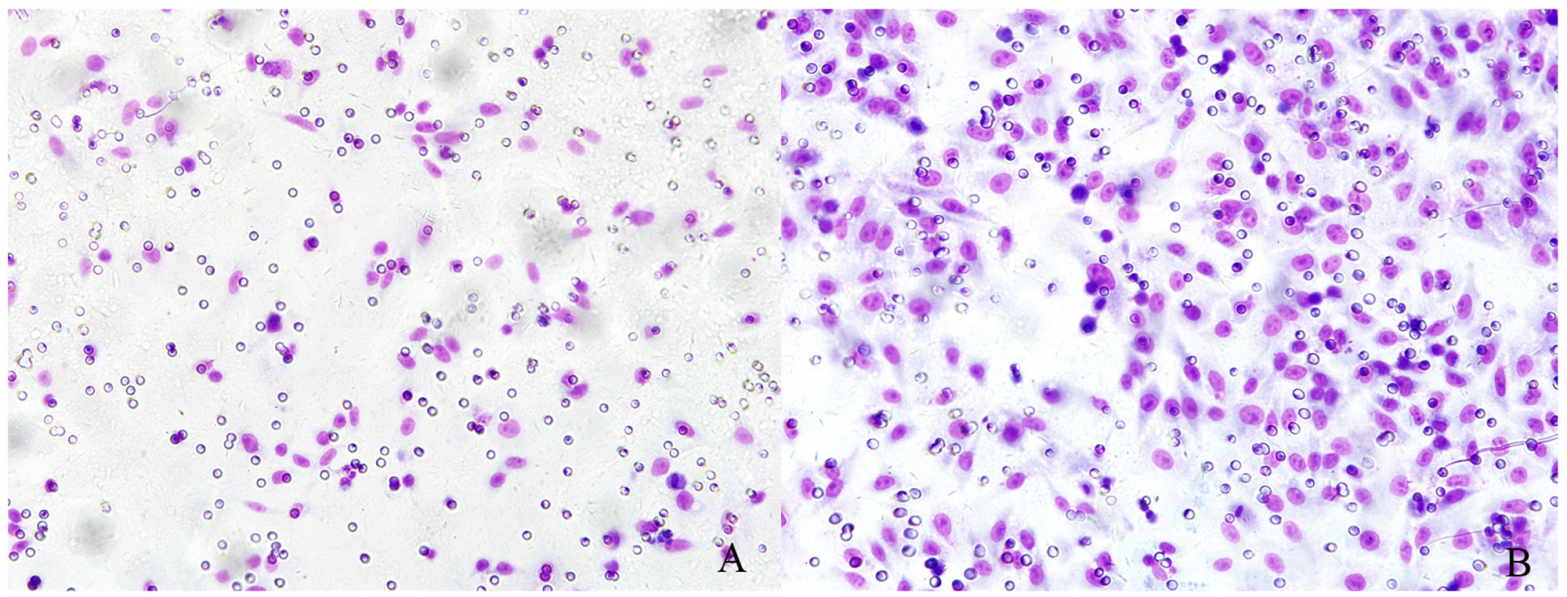
VNP 20009 suppresses migration of McA-RH7777 cells **(A)** VNP 20009 group; **(B)** Control group. The cells passed through the permeable membrane were distinctly fewer in the VNP 20009 groups than those in the control groups.

We performed *in vitro* experiments using the rat hepatocellular carcinoma McA-RH7777 cell line. In the cytotoxicity assays, we observed that the OD values of VNP 20009 groups were obviously lower than those of control groups and decreased as the bacteria concentration increased (Figure 1). VNP 20009 inhibited the proliferation of McA-RH7777 cells. Flow cytometry analyses showed that apoptic cells percentage of VNP 20009 group was much higher than that of control group (Figure 2). VNP 20009 was able to efficiently induce apoptosis of McA-RH7777 cells. In the Transwell migration assay, cells passed through the permeable membrane were distinctly fewer in the VNP 20009 groups than those in the control groups (Figure 3), indicating that VNP 20009 decreased the migratory ability of McA-RH7777 cells. These results revealed that VNP 20009 induced a direct action on the cell viability and mobility leading to a significant reduction in the number of cells and suppression of their invasiveness.

**Figure 4:**
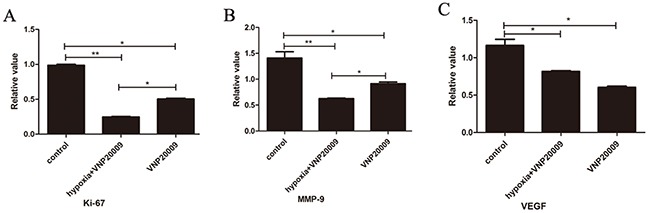
VNP20009 decreases the levels of Ki-67, MMP-9 and VEGF in McA-RH7777 cells assessed by real-time PCR **(A), (B), (C)**: Real-time PCR analyses displayed that Ki-67, MMP-9, and VEGF levels were all higher in the control groups compared with those in VNP 20009 groups (* P < 0.05, ** P < 0.01).

Real-time PCR analyses displayed that Ki-67, MMP-9, and VEGF levels were all higher in the control groups compared with those in VNP 20009 groups (Figure 4). These data also implied that VNP 20009 had the ability of suppressing tumor growth and metastasis.

### Primary tumor growth suppression *in vivo*

**Figure 5:**
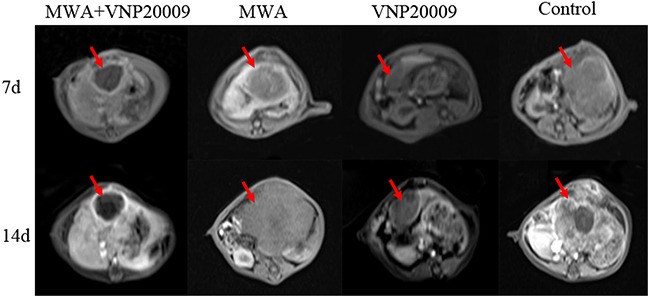
Magnetic resonance imaging (MRI) on the 7th an 14th day after the procedure On the 7th and 14th day post operation, the mean tumor volumes were distinctly smaller in the MWA plus VNP 20009 group than those in other groups. Tumors of both MWA plus VNP 20009 group and VNP 20009 group grew much slower compared with those of MWA group and control group.

On the 7th and 14th day post operation, the mean tumor volumes were distinctly smaller in the MWA plus VNP 20009 group than those in other groups (Table 1, P < 0.05; Figure 5). Notably, the tumors of VNP 20009 group were not obviously eliminated 7 days later compared with those of MWA plus VNP 20009 group and MWA group. On the 14th day, however, the tumors of VNP 20009 group were smaller than those of MWA group. Tumors of both MWA plus VNP 20009 group and VNP 20009 group grew much slower compared with those of MWA group and control group. The results suggested that MWA combined with VNP 20009 had better effects than MWA or VNP 20009 alone in treating HCC. VNP 20009 alone was not able to distinctly extinguish the lesion in short time, but it might play an important role in inhibiting tumor growth.

**Table 1:** Mean tumor volumes of each group on the 7th and 14th day after the procedure

Group	7th Day (mm^3^)	14th Day (mm^3^)
MWA plus VNP20009	22.34 ±2.36	155.32±22.84
MWA	56.32±4.56	6254.47±425.34
VNP 20009	195.23±13.24	922.24±35.63
Control	4024.12 ± 312.68	17820.89 ± 505.36

### Metastasis suppression

**Figure 6:**
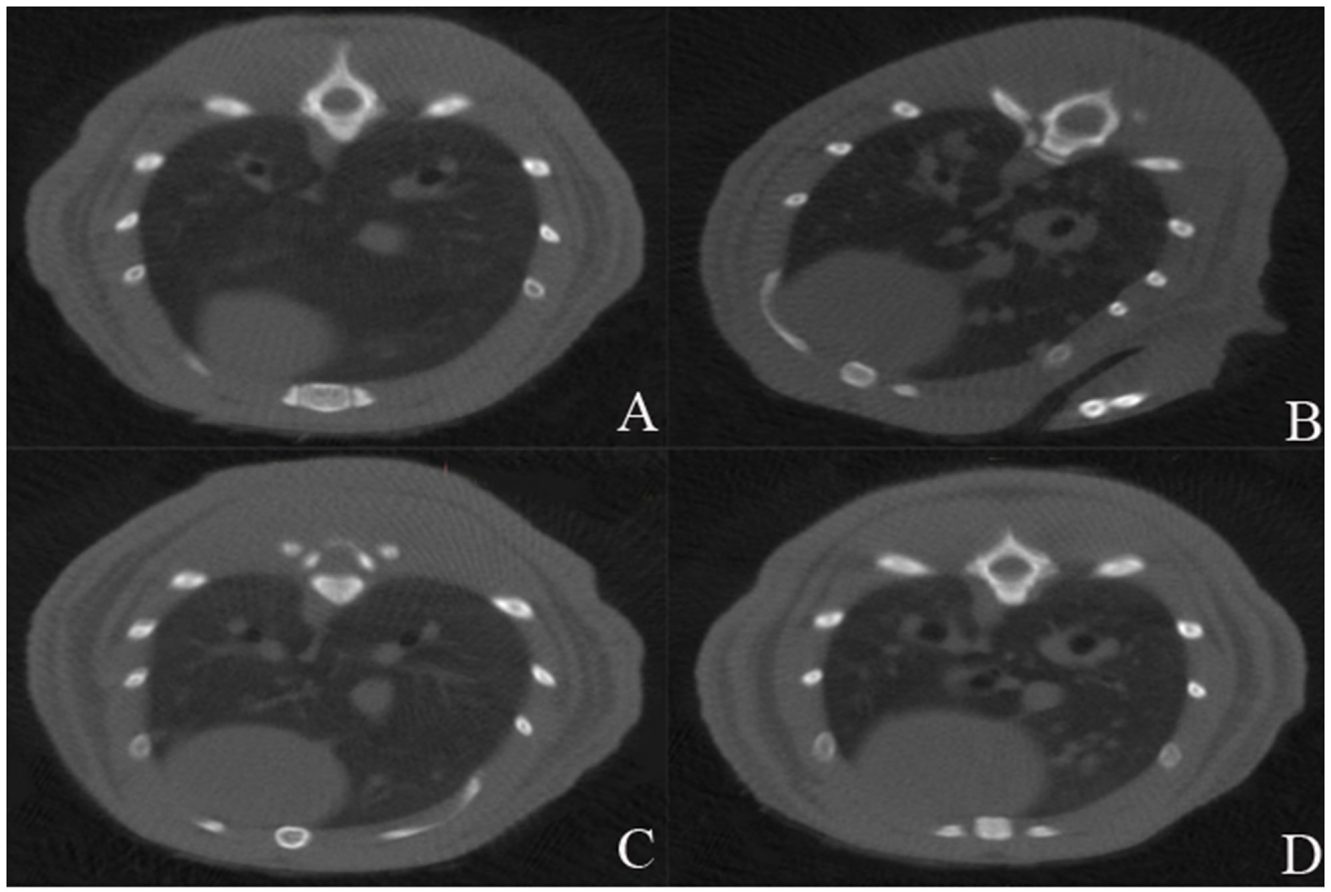
Micro computed tomography(CT) on the 14th day after the procedure **(A)** MWA plus VNP20009 group; **(B)** MWA group; **(C)** VNP 20009 group; **(D)** Control group. The incidences of lung metastases in MWA plus VNP 20009 group and VNP 20009 group were significantly lower than those of MWA group and control group (MWA plus VNP 20009 group 20%, MWA group 60%, VNP 20009 group 30%, and control group 100%, respectively).

The incidences of lung metastases in MWA plus VNP 20009 group and VNP 20009 group were significantly lower than those of MWA group and control group (MWA plus VNP 20009 group 20%, MWA group 60%, VNP 20009 group 30%, and control group 100%, respectively; Figure 6). It implied that VNP 20009 suppressed the lung metastases of tumor individually as well as in conjunction with MWA.

### Survival efficacy

**Figure 7:**
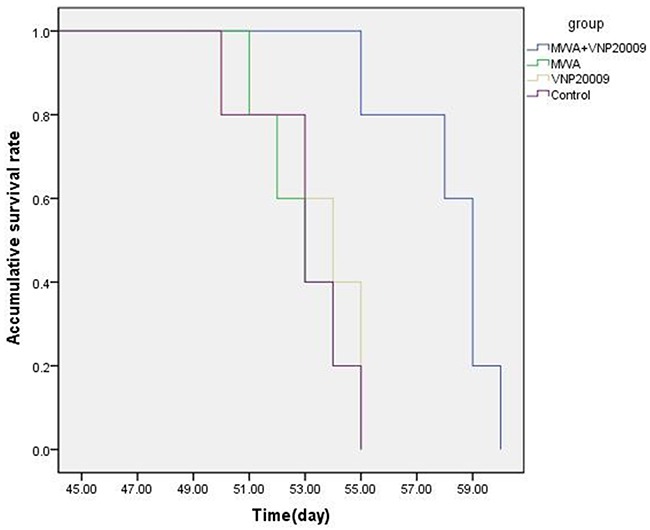
Comparison of the overall survival of rats between groups The median survival time of different groups in our study were as follows: MWA plus VNP 20009 group 58.2 ± 1.92 d, MWA group 53.21±3.73 d, VNP 20009 group 53.59±2.94 d, and control group 52.0 ± 2.71 d, respectively. The Log-rank analysis of survival showed that animals of MWA plus VNP 20009 group survived longer than those of other groups.

The orthotopic HCC models resulted in rapid deaths of animals. The median survival time of different groups in our study were as follows: MWA plus VNP 20009 group 58.2±1.92 d, MWA group 53.21±3.73 d, VNP 20009 group 53.59±2.94 d, and control group 52.0±2.71 d, respectively. The Log-rank analysis of survival showed that animals of MWA plus VNP 20009 group survived longer than those of other groups (P < 0.05, Figure 7). It indicated that VNP 20009 could improve the efficacy of MWA in treating HCC by prolonging survival.

### Bacteria culture, hematoxylin–eosin staining and immunohistochemical analyses

**Figure 8:**
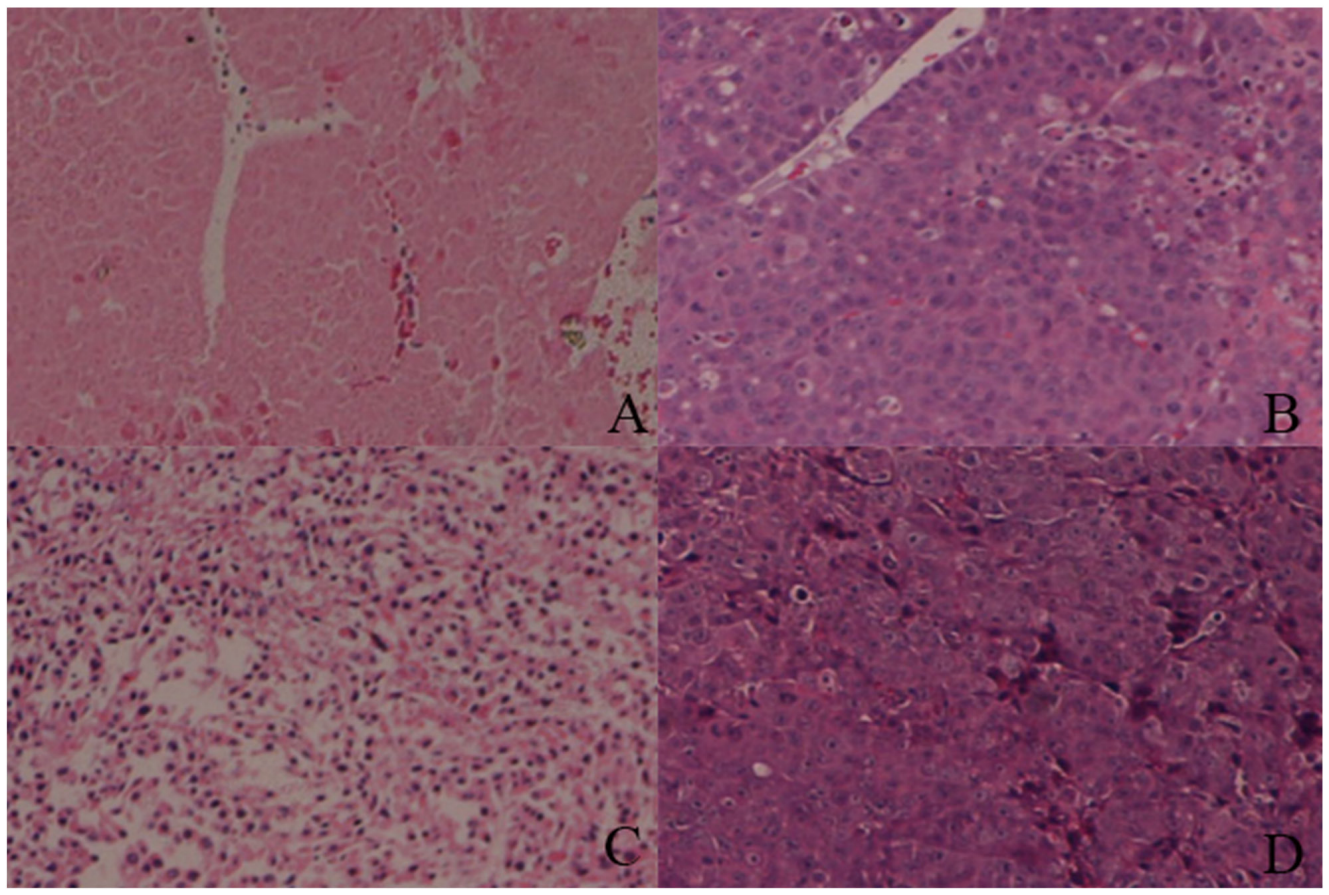
Hematoxylin–eosin (H&E) staining for tumor tissues **(A)** MWA plus VNP20009 group; **(B)** MWA group; **(C)** VNP 20009 group; **(D)** Control group. The interior of tumors of MWA plus VNP 20009 group were mainly necrosis, while lots of cancer cells were observed in tumors of other groups. Hematoxylin-eosin, magnification 10×.

**Figure 9:**
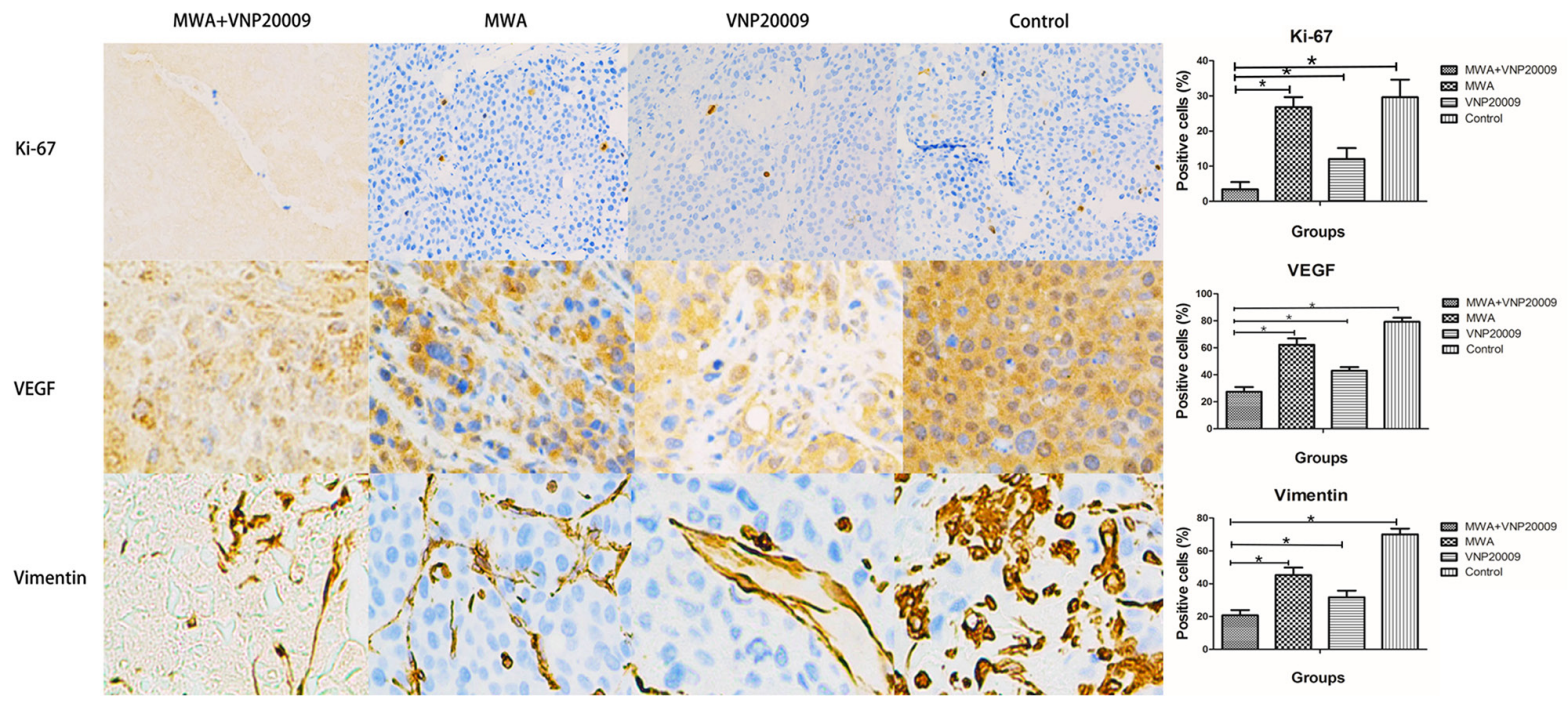
Immunohistochemical analyses for tumor tissues The levels of Ki-67, VEGF and Vimentin in the tumors of MWA plus VNP20009 group were significantly lower than those of other groups (* P < 0.05). Immunohistochemical analyses, ×10.

The results of bacteria culture displayed that the anaerobia in tumors were much more than those in other organs. It implied that VNP 20009 treatment is a safe option. HE staining demonstrated that the interior of tumors of MWA plus VNP 20009 group were mainly necrosis, while lots of cancer cells were observed in tumors of other groups (Figure 8). Immunohistochemical analyses revealed that the levels of Ki-67, VEGF and Vimentin in the tumors of MWA plus VNP20009 group were significantly lower than those of other groups (P < 0.05; Figure 9). Ki-67, VEGF and Vimentin were well-known markers in relation to tumor growth and metastasis. Thus, it could be inferred that VNP 20009 was able to suppress tumor growth and metastasis.

## DISCUSSION

In this study, we investigated a therapy strategy, MWA combined with VNP 20009, to treat a rat model of HCC. MWA is known to be an effective and less invasive modality in treating tumors, which can completely eradiate eligible early-stage tumors [6, 17, 18]. *Salmonella typhimurium* VNP 20009, as a tumor-targeting facultative anaerobia, had been proved to have antitumor effect on multiple tumor models [16, 19]. In contrast to obligate anaerobia such as *Clostridia* and *bifidobacterium*, VNP20009 can grow under both anaerobic and aerobic conditions. So it is able to colonize hypoxic or necrotic tumor areas as well as well-oxygenated tumor tissues [20]. In our work, MWA combined with VNP20009 produced better therapeutic effects than either MWA or VNP20009 alone. MWA was able to successfully diminish the tumor volume so as to decrease the burden of tumors promptly. Although VNP20009 therapy seemed not to obviously eliminate the tumor in short time, it successfully inhibited tumor growth either as a single agent or combined with MWA.

The animals in the MWA plus VNP 20009 group and VNP 20009 group had lower rates of lung metastases in our study. It implied that VNP 20009 had the potential to suppress metastases and decrease the degree of tumor malignancy, which was consistent with previous report [21].

Notably, the levels of Ki-67, VEGF and Vimentin in the tumors of MWA plus VNP20009 group were also the lowest in all groups. Ki-67, VEGF and Vimentin were known to be associated with tumor growth, metastasis or differentiation. This might, at least in part, accounted for the good antitumor effects produced by MWA combined with VNP 20009 therapy. Moreover, it was reported that bacteria treatment could stimulate a specific immune pattern and a potent inflammatory response, which could induce tumor-specific immune responses and contrast the immunosuppressive environment generated by tumors [22, 23].

The primary advantages of VNP 20009 as an antitumor agent are safety profiles and efficient results which had been proved in preclinical and clinical trials [12, 24]. The outcome of bacteria culture for tumors and other organs in this study showed that the bacteria preferentially concentrated in tumors, confirming the safety of VNP 20009 therapy. Several interacting mechanisms were supposed to control the accumulation of bacteria in tumors: flooding into tumors following inflammation [25]; entrapment of bacteria in the irregular vasculature of tumors [26]; chemotaxis towards compounds released by tumors [27]; protection from clearance by the immune system [28]; and preferential growth in tumor-specific microenvironment [29].

The limitations in our study include relatively short follow-up period, small number of subjects, and lack of investigation on the immunological mechanisms of VNP20009 treatment. Inevitably, the limitations may to some degree affect the reliability of our conclusion. In the future, more attention should be paid on the research of immunological mechanisms and genetic engineering of VNP 20009 so as to make it a more powerful tumor-target tool.

## CONCLUSION

MWA combined with VNP 20009 was a safe and effective strategy in treating HCC, producing better results than MWA or VNP 20009 alone. VNP 20009 therapy was able to successfully inhibit the tumor growth and suppress tumor metastases.

## MATERIALS AND METHODS

### Cell line and bacteria

McA-RH7777, a kind of Buffalo rat hepato-cellular carcinoma cell line, was obtained from the American Type Culture Collection (no. CRL1601; ATCC, Manassas, VA, USA), and was cultured in Dulbecco's Modified Eagle's Medium (DMEM) supplemented with 10% fetal bovine serum (Thermo Fisher Scientific) and 1% streptomycin and penicillin (Thermo Fisher Scientific, Waltham, MA, USA). The cell line was kept at 37°C in 5% CO_2_. *Salmonella typhimurium* strain VNP 20009 (ATCC, Manassas, VA, USA) was cultured and prepared as described [15].

### Animals

Buffalo rats (Charles River Laboratories, Wilmington, MA, USA) were bred in Experimental Animal Research Center at Shanghai Medical School, Fudan University. All animal experiments were ethically approved by Fudan University. Only male rats weighed 150~200 g were selected for the experiments.

### *Salmonella* cytotoxicity assay

*Salmonella* cytotoxicity on McA-RH7777 cells was measured by cell counting kit-8 (CCK-8) (Dojindo, Kumamoto, Japan) cytotoxic assay. McA-RH7777 cells were seeded in 96-well plates to a density of 1×10^4^ cells per well in 200 μl DMEM with 10% FBS. VNP 20009 was diluted in DMEM and added to McA-RH7777 cells at a density of 1×10^5^, 1×10^6^, or 1×10^7^ per well, respectively. Then the cells were incubated at 37°C in 5% CO_2_. CCK-8 solution (10 μl/well) was added and incubated for 2 h. The absorbance at 450 nm were measured.

### Flow cytometry analysis of cell apoptosis induced by VNP 20009

Analysis of McA-RH7777 cell apoptosis induced by VNP 20009 was conducted by flow cytometry (BD, Franklin Lakes, USA). McA-RH7777 cells were incubated with VNP 20009 (1×10^5^, 1×10^6^, or 1×10^7^ per well) for 1 hour in the medium without antibiotics. Then the cells were washed with PBS and incubated in DMEM supplemented with 50 μg/ml gentamicin for 24 hours to kill merely extracellular bacteria. After staining with 50 μg/ml propidium iodide and FITC-Annexin V (BD, Franklin Lakes, USA), cells were analyzed by flow cytometry to evaluate apoptosis.

### Transwell migration assay

McA-RH7777 cells were seeded in 24-well plates to a density of 1×10^4^ cells per well in the appropriate medium and incubated with VNP 20009 (1×10^6^ per well) for 1 hour. Cells were rinsed with PBS and incubated in a medium containing gentamicin (50 μg/ml) for 48 hours to kill only extracellular bacteria. Transwell chamber (Corning, USA) was used to study the migratory response of McA-RH7777 cells to VNP 20009 according to the instructions.

### Real-time PCR analysis

McA-RH7777 cells were cultured and treated with bacteria as above mentioned. CoCl_2_ was used to create a hypoxic environment. Total RNAs were extracted from infected cells using TRIzol reagent (Invitrogen, Carlsbad, USA) and were reverse transcribed into cDNA using a M-MLV qPCR Kit (Invitrogen, Carlsbad, USA). The sequences of the antisense and sense primers were displayed in Table 2.

**Table 2:** Real-time PCR markers and sequences

Marker	Sequence
VEGF-1	AGTCTGTGCTCTGGGATTTG
VEGF-2	GGTCTCTCTCTCTCTCTCTCTTC
MMP9-1	CGCCAACTATGACCAGGATAAG
MMP9-2	GTTTAGAGCCACGACCATACAG
Ki67-1	CACCACCAGAGCCAATAGATAC
Ki67-2	CTGTGTCCAATTTCCGCTTTAC
rat actin-F	AGTGTGACGTTGACATCCGT
rat actin-R	GACTCATCGTACTCCTGCTT

### Surgical orthotopic implantation of HCC

The Buffalo rats were kept under anesthesia during the operation. Tumor fragments (1 mm^3^) were prepared from a McA-RH7777 tumor growing subcutaneously in a Baffalo rat. One tumor fragment was implanted by surgical orthotopic implantation in the left lobe of the liver exposed after an upper midline abdominal incision. The incision in the abdominal wall was closed with a 6–0 surgical suture in two layers. All procedures of the surgery were performed with a ×7 magnification microscope (Nikon, Tokyo, Japan).

### Incomplete microwave ablation and bacterial infection

Three weeks after the establishment of tumor models, forty rats were randomly divided into four groups (10 rats each group): (1) MWA plus VNP 20009 group; (2) MWA group; (3) VNP 20009 group; and (4) control group. The procedure of MWA was performed by using an ECO-100C microwave generator (ECO Medical Equipment Co Ltd, Jiangsu, China). After proper exposure of the hepatic carcinoma, special electrode was inserted into the tumor but not reach out the farthest margin in order to achieve incomplete necrosis. Energy delivery was applied at 20 W for 2–3 minutes. Multiple ablations were performed if necessary. For infection of VNP20009, the bacteria (1×10^7^ cfu/rat) were administered with 0.2 ml PBS by the means of intra-tumor injection following MWA. The control group received sham operations. Animals were kept under anesthesia during all the procedures described above.

### Analysis of antitumor efficacy

MRI (3.0T, Magnetom avanto, Siemens, Germany) scans (T1WI, T2WI, DWI and DCE-MRI) were performed before operations to assess the tumor profile. Tumor responses were evaluated by the method of MRI one and two weeks after treatment, respectively. The tumor volumes were calibrated according to the following formula: (length × width^2^)/2 [30]. Micro CT scans were used to observe the lung metastases on the 14th day post operation. Half of rats each group were sacrificed following the MRI and micro CT scans two weeks after treatment, left animals were followed until death. Survival time were recorded.

### Bacteria culture, hematoxylin–eosin staining and immunohistochemical analysis

After the animals were sacrificed or died, the tumors were cut off. Part of tumor tissues were fixed with 4% formaldehyde, embedded in paraffin and sectioned for hematoxylin–eosin (H&E) staining and immunohistochemical analysis according to standard histological procedures.

### Statistical analysis

All values in the work were expressed as mean ± SE. Comparison between groups were processed by using the unpaired Student's *t*-test after proving the assumption of normality. The overall survival was evaluated by using the Kaplan-Meier method. The tests were performed by using the Statistical Package for the Social Sciences (SPSS) program (version 20.0; IBM Corporation, Armonk, NY, USA). The criterion for significance was stipulated as P < 0.05.

## References

[1] Forner A, Llovet JM, Bruix J (2012). Hepatocellular carcinoma. Lancet.

[2] Qian G, Wang N, Shen Q, Sheng YH, Zhao J, Kuang M, Liu G, Wu M (2012). Efficacy of microwave versus radiofrequency ablation for treatment of small hepatocellular carcinoma: experimental and clinical studies. Eur Radiol.

[3] Liang P, Wang Y, Yu X, Dong B (2009). Malignant Liver Tumors: Treatment with Percutaneous Microwave Ablation--Complications among Cohort of 1136 Patients1. Radiology.

[4] Zhou F, Yu X, Liang P, Cheng Z, Han Z, Yu J, Liu F, Tan S, Dai G, Bai L (2016). Combined microwave ablation and systemic chemotherapy for liver metastases from oesophageal cancer: Preliminary results and literature review. Int J Hyperthermia.

[5] Yang G, Zhao Q, Qian S, Zhu L, Qu X, Zhang W, Yan Z, Cheng J, Liu Q, Liu R, Wang J (2015). Percutaneous microwave ablation combined with simultaneous transarterial chemoembolization for the treatment of advanced intrahepatic cholangiocarcinoma. Oncotargets Ther.

[6] Liu C, Liang P, Liu F, Wang Y, Li X, Han Z, Liu C (2011). MWA Combined with TACE as a combined therapy for unresectable large-sized hepotocellular carcinoma. Int J Hyperther.

[7] Ni J, Sun H, Chen Y, Luo J, Chen D, Jiang X, Xu L (2014). Prognostic factors for survival after transarterial chemoembolization combined with microwave ablation for hepatocellular carcinoma. World J Gastroentero.

[8] McCarthy EF (2006). The toxins of William B. Coley and the treatment of bone and soft-tissue sarcomas. Iowa Orthop J.

[9] Yin X, Yu B, Tang Z, He B, Ren J, Xiao X, Tang W (2012). Bifidobacterium infantis-mediated HSV-TK/GCV suicide gene therapy induces both extrinsic and intrinsic apoptosis in a rat model of bladder cancer. Cancer Gene Ther.

[10] Talib W, Mahasneh A (2012). Combination of Ononis hirta and Bifidobacterium longum decreases syngeneic mouse mammary tumor burden and enhances immune response. J Cancer Res Ther.

[11] Nuyts S, Van Mellaert L, Theys J, Landuyt W, Bosmans E, Anne J, Lambin P (2001). Radio-responsive recA promoter significantly increases TNFalpha production in recombinant clostridia after 2 Gy irradiation. Gene Ther.

[12] Toso JF, Gill VJ, Hwu P, Marincola FM, Restifo NP, Schwartzentruber DJ, Sherry RM, Topalian SL, Yang JC, Stock F, Freezer LJ, Morton KE, Seipp C (2002). Phase I study of the intravenous administration of attenuated Salmonella typhimurium to patients with metastatic melanoma. J Clin Oncol.

[13] Yam C, Zhao M, Hayashi K, Ma H, Kishimoto H, McElroy M, Bouvet M, Hoffman RM (2010). Monotherapy with a tumor-targeting mutant of *S. typhimurium inhibits* liver metastasis in a mouse model of pancreatic cancer. J Surg Res.

[14] Yu B, Yang M, Shi L, Yao Y, Jiang Q, Li X, Tang LH, Zheng BJ, Yuen KY, Smith DK, Song E, Huang JD (2012). Explicit hypoxia targeting with tumor suppression by creating an "obligate" anaerobic Salmonella Typhimurium strain. Sci Rep.

[15] Low KB, Ittensohn M, Luo X, Zheng LM, King I, Pawelek JM, Bermudes D (2004). Construction of VNP20009: a novel, genetically stable antibiotic-sensitive strain of tumor-targeting Salmonella for parenteral administration in humans. Methods Mol Med.

[16] Jia LJ, Wei DP, Sun QM, Huang Y, Wu Q, Hua ZC (2007). Oral delivery of tumor-targeting Salmonella for cancer therapy in murine tumor models. Cancer Sci.

[17] Abdelaziz A, Elbaz T, Shousha HI, Mahmoud S, Ibrahim M, Abdelmaksoud A, Nabeel M (2014). Efficacy and survival analysis of percutaneous radiofrequency versus microwave ablation for hepatocellular carcinoma: an egyptian multidisciplinary clinic experience. Surgical Endoscopy.

[18] Ding J, Jing X, Liu J, Wang Y, Wang F, Wang Y, Du Z (2013). Comparison of two different thermal techniques for the treatment of hepatocellular carcinoma. Eur J Radiol.

[19] Chen G, Wei DP, Jia LJ, Tang B, Shu L, Zhang K, Xu Y, Gao J, Huang XF, Jiang WH, Hu QG, Huang Y, Wu Q (2009). Oral delivery of tumor-targeting Salmonella exhibits promising therapeutic efficacy and low toxicity. Cancer Sci.

[20] Lee CH (2012). Engineering bacteria toward tumor targeting for cancer treatment: current state and perspectives. Appl Microbiol Biotechnol.

[21] Sorenson BS, Banton KL, Frykman NL, Leonard AS, Saltzman DA (2008). Attenuated Salmonella typhimurium with interleukin 2 gene prevents the establishment of pulmonary metastases in a model of osteosarcoma. J Pediatr Surg.

[22] Chirullo B, Ammendola S, Leonardi L, Falcini R, Petrucci P, Pistoia C, Vendetti S, Battistoni A, Pasquali P (2015). Attenuated mutant strain of Salmonella Typhimurium lacking the ZnuABC transporter contrasts tumor growth promoting anti-cancer immune response. Oncotarget.

[23] Avogadri F, Martinoli C, Petrovska L, Chiodoni C, Transidico P, Bronte V, Longhi R, Colombo MP, Dougan G, Rescigno M (2005). Cancer immunotherapy based on killing of Salmonella-infected tumor cells. Cancer Res.

[24] Heimann DM, Rosenberg SA (2003). Continuous intravenous administration of live genetically modified Salmonella typhimurium in patients with metastatic melanoma. J Immunother.

[25] Leschner S, Westphal K, Dietrich N, Viegas N, Jablonska J, Lyszkiewicz M, Lienenklaus S, Falk W, Gekara N, Loessner H, Weiss S (2009). Tumor invasion of Salmonella enterica serovar Typhimurium is accompanied by strong hemorrhage promoted by TNF-alpha. Plos One.

[26] Forbes NS, Munn LL, Fukumura D, Jain RK (2003). Sparse initial entrapment of systemically injected Salmonella typhimurium leads to heterogeneous accumulation within tumors. Cancer Res.

[27] Kasinskas RW, Forbes NS (2006). Salmonella typhimurium specifically chemotax and proliferate in heterogeneous tumor tissue in vitro. Biotechnol Bioeng.

[28] Sznol M, Lin SL, Bermudes D, Zheng L, King I (2000). Use of preferentially replicating bacteria for the treatment of cancer. J Clin Invest.

[29] Zhao M, Yang M, Li XM, Jiang P, Baranov E, Li S, Xu M, Penman S, Hoffman RM (2005). Tumor-targeting bacterial therapy with amino acid auxotrophs of GFP-expressing Salmonella typhimurium. Proc Natl Acad Sci U S A.

[30] Mendez O, Zavadil J, Esencay M, Lukyanov Y, Santovasi D, Wang SC, Newcomb EW, Zagzag D (2010). Knock down of HIF-1alpha in glioma cells reduces migration in vitro and invasion in vivo and impairs their ability to form tumor spheres. Mol Cancer.

